# Evaluation of *Limosilactobacillus reuteri* ATCC PTA 6127 Reveals Multilayered Antimicrobial and Epithelial Barrier-Supportive Effects in a Canine Epithelial Model

**DOI:** 10.3390/microorganisms14071422

**Published:** 2026-06-29

**Authors:** Josh Walker, Akila Rekima, Andreea Cornelia Udrea, Katrine Bie Larsen, Adrian Schwarzenberg, Steffen Yde Bak, Niels Christensen, Svetlana Gerdes, Weiqing Zeng, Ashley Hibberd, Chong Shen

**Affiliations:** 1 Direct-Fed Microbials Laboratory, R&D, Health & Biosciences, International Flavors & Fragrance (IFF), Nutrition Biosciences USA 1, LLC, 200 Powder Mill Road, Experimental Station—E361, Wilmington, DE 19803, USA; joshua.walker@iff.com (J.W.); weiqing.zeng@iff.com (W.Z.); 2 Gut Immunology Laboratory, R&D, Health & Biosciences, International Flavors & Fragrance (IFF), Edwin Rahrs Vej 38, 8220 Brabrand, Denmark; akila.rekima@iff.com (A.R.); andreea.cornelia.udrea@iff.com (A.C.U.); katrinebie.larsen@iff.com (K.B.L.); 3 Enabling Technologies, R&D, Health & Biosciences, International Flavors & Fragrance (IFF), Edwin Rahrs Vej 38, 8220 Brabrand, Denmarksteffen.yde.bak@iff.com (S.Y.B.); niels.christensen@iff.com (N.C.); 4 R&D, Health & Biosciences, International Flavors & Fragrance (IFF), Madison, WI 53716, USAashley.hibberd@iff.com (A.H.)

**Keywords:** *Limosilactobacillus reuteri*, canine epithelial cells, enteric pathogens, antimicrobial activity, epithelial wound healing

## Abstract

Good canine gastrointestinal health depends on the suppression of enteric pathogens and maintenance of epithelial barrier integrity. *Limosilactobacillus reuteri* ATCC PTA 6127 (Lr6127) is a dog-derived probiotic, but evidence supporting its functional properties remains limited. Here, we evaluated the antimicrobial and epithelial-supportive effects of Lr6127 using a canine epithelial cell model. Cell-free supernatant (CFS) from Lr6127 significantly inhibited the growth of canine-relevant pathogens, including Enterotoxigenic *Escherichia coli* (52.0 ± 1.3%), *Clostridium perfringens* (54.0 ± 2.7%), and *Salmonella enterica* subsp. *enterica* serovar Typhimurium (48.6 ± 1.2%), compared with the medium control (*p* < 0.0001). Pathogen inhibition increased in a dose-dependent manner with increasing CFS concentration. Untargeted metabolomic analysis revealed enrichment of multiple antimicrobial-associated metabolites, indicating a multi-component profile consistent with pathogen suppression, with genomic analysis supporting the aromatic amino acid-derived metabolite findings. In addition, viable Lr6127 significantly reduced the epithelial adhesion of all the tested pathogens (*p* < 0.01). Beyond direct antimicrobial effects, Lr6127 CFS promoted epithelial wound healing at later time points, accompanied by the coordinated modulation of proteins associated with cytoskeletal remodeling and barrier repair. Collectively, these findings support the idea that Lr6127 is associated with antimicrobial and epithelial-related effects, highlighting its potential to contribute to epithelial function under controlled in vitro conditions.

## 1. Introduction

The canine gastrointestinal tract plays a central role in nutrient absorption, immune surveillance, and protection against enteric pathogens. Disruption of this ecosystem—through dietary transitions, stress, antibiotic exposure, or infection—commonly leads to gastrointestinal disease, which manifests as acute or chronic diarrhea, mucosal inflammation, and compromised barrier function [1]. Enteric bacterial pathogens are a major contributor to these disorders in dogs, both in clinical practice and under conditions of environmental or dietary challenge [1].

Among these pathogens, Enterotoxigenic *Escherichia coli* (ETEC), *Clostridium perfringens* (*C. perfringens*), and *Salmonella enterica* subsp. *enterica* serovar Typhimurium (*S.* Typhimurium) represent clinically and mechanistically distinct threats to canine gut health. Despite their differences, they converge in their ability to colonize to disrupt epithelial function and compromise intestinal barrier integrity [1,2,3]. These pathogens are able to colonize the gastrointestinal tract [4], adhere to epithelial surfaces [5], and disrupt barrier integrity [6]. Successful infection is initiated by bacterial colonization of the host [7], which requires adaptation to the gut environment [8] and interaction with epithelial surfaces [9]. A critical step in this process is bacterial adhesion to epithelial cells [5], which enables persistence [10], niche establishment [7], and resistance to mechanical and host-mediated clearance [10]. Adhesion represents one of the earliest events in host–pathogen interactions [11] and directly influences infection outcome [7]. These early interactions can alter epithelial barrier function [12], disrupt tight junction integrity [13], and impair host defense mechanisms [14].

Probiotic microorganisms, particularly strains of *Limosilactobacillus reuteri* (*L. reuteri*), have been extensively investigated for their ability to modulate gut microbial ecology [15] and support epithelial homeostasis [16]. *L. reuteri* exhibits antimicrobial activity [17] and metabolic versatility [15], and it also interacts directly with host epithelial systems [18]. Strains of *L. reuteri* contribute to maintaining intestinal barrier integrity [16] through the competitive exclusion of pathogens [19] and modulation of microbial composition [15]. *L. reuteri* can also regulate epithelial barrier function and tight junction protein expression [13].

A defining feature of *L. reuteri* is the production of bioactive metabolites [15]. Among these, reuterin, a broad-spectrum antimicrobial compound derived from glycerol metabolism, plays a central role [20]. Reuterin has been shown to inhibit a wide range of pathogens [17] while modulating microbial community structure [20]. Accordingly, the antimicrobial activity of *L. reuteri* is closely linked to its metabolic profile [17]. In addition to direct antimicrobial effects, probiotic-derived metabolites and secreted factors can influence epithelial cell behavior [21]. These effects include the modulation of cell migration [21] and proliferation [22], both of which are critical processes in epithelial restitution [22] and wound healing [21]. Consequently, probiotic-derived compounds can promote re-epithelialization and support tissue repair [22]. Importantly, probiotic functionality is highly strain-specific [23], meaning that beneficial effects cannot be generalized across species [24]. Distinct strains exhibit different metabolic and functional properties [16], necessitating targeted evaluation at the individual strain level [23].

*Limosilactobacillus reuteri* ATCC PTA 6127 (Lr6127) is a host-derived strain of canine origin [25]. It has been used in applied and commercial formulations [25]. Host-associated *L. reuteri* strains exhibit ecological adaptation [16] that may influence their functional properties [15]. However, the mechanistic interaction of Lr6127 with epithelial systems remains insufficiently characterized. Its effects on pathogen growth are not well defined, its influence on epithelial adhesion has not been systematically studied, and its role in epithelial repair processes remains unclear. This represents a critical knowledge gap in strain-specific probiotic research [23].

Recent studies highlight the importance of evaluating cell-free supernatants [21], which contain bioactive metabolites [22]. These components can exert antimicrobial activity [21] and modulate epithelial responses [22]. They also contribute to epithelial repair processes [21] and wound healing [22]. Therefore, the objective of the present study was to systematically evaluate the functional properties of Lr6127 using an in vitro canine epithelial model. Specifically, the study was designed to (i) characterize bacterial growth behavior and antimicrobial activity, (ii) assess adhesion to epithelial cells in the context of pathogen interaction, and (iii) determine the effects of cell-free supernatants on epithelial wound healing as a measure of epithelial restitution.

## 2. Materials and Methods

### 2.1. Reagents and Materials

All the cell culture media, equipment, and reagents were purchased from Thermo Fisher Scientific (Roskilde, Denmark) unless otherwise stated.

### 2.2. Bacterial Strains, Culture Conditions, and Cell-Free Supernatant Preparation

The probiotic strain *Limosilactobacillus reuteri* (ATCC PTA-6127; PureStrong™, Lr6127) was characterized by and licensed from BioGaia AB (Stockholm, Sweden). ETEC, *C. perfringens*, and *S.* Typhimurium were obtained from the Danisco Global Culture Collection (DGCC; Niebüll, Germany). The species identification and genetic characterization are summarized in Supplementary Table S1.

Lr6127 was cultured with de Man–Rogosa–Sharpe (MRS) agar and broth at 37 °C. *C. perfringens* pathogenic strains were grown on Brain–Heart Infusion (BHI) agar and in Reinforced Clostridial Medium + 0.1% Sodium Resazurin (RCM) broth at 37 °C under anaerobic conditions using Oxoid AnaeroGen sachets. ETEC and *S.* Typhimurium were cultured on Tryptic Soy Agar (TSA) and Broth (TSB) at 37 °C under aerobic conditions.

Cell-free supernatant (CFS) was prepared from Lr6127. Lr6127 colonies were grown from glycerol freezer stocks by streaking on agar followed by overnight incubation. Individual colonies were collected and resuspended by pipetting in MRS medium in a 2 mL microtube, allowing large particulates to settle. The upper fraction of the suspension was transferred to a new container, and the optical density was measured at 600 nm (OD_600_). All the optical density measurements were performed using a Synergy MX microplate reader (BioTek Instruments, Inc., Highland Park, VT, USA). The volume of this suspension equivalent to 50 μL with an OD_600_ of 1 was calculated using the following formula:

x volume of suspension required = 50 μL/(OD_x_ − OD_y_)

where

OD_x_ = OD600 of cell suspension;

OD_y_ = OD_600_ of culture medium alone.

This approximation assumes that the OD decreases linearly with dilution. Only OD_x_ values below 1.5 were accepted; cultures exceeding this threshold were diluted with additional fresh medium and remeasured. The calculated suspension volume was used to inoculate 25 mL of the appropriate broth in a 125 mL flat-bottom flask. Cultures were incubated for 6 h, after which the OD_600_ was measured again. The culture volume equivalent to 10 uL of culture with an OD_600_ of 1 was calculated using the same approach by substituting 50 µL with 10 µL in the formula. This volume was used to inoculate 50 mL of broth in a 250 mL flat-bottom flask, which was incubated with gentle shaking for 18 h. The culture was then centrifuged at 5000× *g* for 5 min, and the resulting supernatant was sterilized by vacuum filtration through a 0.2 µm filter (500 mL, aPES membrane, 75 mm diameter, #566-0020, Thermo Scientific Nalgene, New York, NY, USA) to generate CFS. CFS preparations were stored at −20 °C until use.

### 2.3. Epithelial Cell Culture and CFS Treatment

The canine proximal epithelial cell line MCA-B1 (ACC 828, DSMZ; Braunschweig, Germany) was propagated in DMEM/F12 medium supplemented with 10% heat-inactivated fetal bovine serum (FBS) and 1% penicillin–streptomycin (100 U/mL penicillin, 100 µg/mL streptomycin). CFS was added to cell cultures at a maximum concentration of 5% (*v*/*v*) and cultured overnight at 37 °C in a 5% CO_2_ environment. Prior testing confirmed that this did not alter the final medium pH, which remained between 7.0 and 7.2. The optical density values were correlated with colony-forming units (CFUs) through serial dilution and plate counts (OD_600_ = 1.0 corresponding to approximately 1 × 10^9^ CFU/mL). All experiments used CFS at a working concentration equivalent to secretions from 1 × 10^7^ CFU/mL. With respect to control conditions, a “medium control” containing equivalent basal medium was used. Because the Lr6127 CFS was derived in MRS and applied at a defined proportion, the control included the same volume of fresh MRS to account for medium-related effects.

### 2.4. Pathogen Inhibition Assay

ETEC, *C*. *perfringens*, and *S.* Typhimurium isolates were obtained from the Danisco Global Culture Collection and used for pathogen inhibition and epithelial interaction assays. Isolates were selected based on their toxin profiles and virulence-associated characteristics rather than host origin. Key pathogenic mechanisms relevant to epithelial interactions, including toxin-mediated epithelial disruption, are conserved across host species, supporting the use of isolates with comparable toxin profiles in the present canine epithelial cell model. Species identification, available host origin, country of isolation, and virulence characteristics are summarized in Supplementary Table S1. Colonies of pathogenic bacteria were resuspended in duplicate in 200 µL of the appropriate broth in 96-well microplates (Corning #3474) and incubated at 37 °C with shaking for 4 h. These preliminary cultures were used to inoculate duplicate assay plates containing the pathogen’s growth medium supplemented with Lr6127 CFS. Wells containing no CFS served as medium controls (0% CFS). For the ETEC and *S.* Typhimurium assays, wells contained 20% (*v*/*v*) CFS and 1% (*v*/*v*) inoculum, whereas the *C. perfringens* assays used 20% (*v*/*v*) CFS and 4% (*v*/*v*) inoculum. The final volume per well was 200 µL. For the dose–response assays, assays were set up with CFS concentrations of 0%, 5%, 10%, 15%, and 20% (corresponding to 0, 10, 20, 30, and 40 μL). For conditions under 20% CFS, the remaining volume was replaced with MRS so that each condition had a total of 20% MRS and/or CFS (spent MRS). All plates were incubated at 37 °C for 16 h with shaking at 200 rpm, after which the OD_600_ was measured, and the percentage growth inhibition was calculated using the following equation:

% inhibition = [(1 − (OD_a_ − OD_0_)/(OD_b_ − OD_0_))] × 100

where

OD_a_ = OD_600_ of medium containing pathogen inoculum and probiotic CFS (challenge well);

OD_0_ = OD_600_ of medium without inoculum or probiotic CFS (negative control);

OD_b_ = OD_600_ of medium containing pathogen inoculum but without probiotic CFS (unchallenged/medium control).

Experiments were performed three times with three replicates per experiment. For statistical analysis, technical replicates were averaged within each experiment, and isolate-level means were used as independent observations.

As a quality control step, absorbance measurements from isolates exhibiting insufficient growth—defined as an OD_600_ < 0.5 in the medium control—were excluded from further analysis. Outliers were identified using the interquartile range (IQR) method, with values exceeding Q3 + 1.5 × IQR or below Q1 − 1.5 × IQR removed from the dataset. Following QC filtering, the mean percentage inhibition values were calculated for 8 *Escherichia coli*, 9 *Clostridium perfringens*, and 5 *S.* Typhimurium isolates.

### 2.5. Untargeted Metabolomics of CFSs

Lr6127 cell-free supernatant (CFS) was generated as previously described, with MRS medium cultured under identical conditions serving as the medium control. Samples were randomized and diluted in a 1:1 ratio with H_2_O:MeOH using an Opentrons Flex automated liquid-handling system (Opentrons Labworks, Inc., Long Island City, NY, USA). A pooled quality control (QC) sample was created by combining aliquots from all experimental samples and was analyzed after every ten injections.

Metabolomic analysis was conducted using a 1290 Infinity III UHPLC system (Agilent Technologies, Glostrup, Denmark) coupled to a timsTOF Flex MALDI 2 mass spectrometer (Bruker, Roskilde, Denmark). Chromatographic separation was performed on an Acquity UPLC HSS T3 column employing a water–acetonitrile gradient containing 0.1% formic acid. Samples were injected at a volume of 5 µL with a flow rate of 0.25 mL/min. Data acquisition was carried out in both positive and negative ionization modes using a VIP HESI source, with MS/MS spectra collected via parallel accumulation–serial fragmentation (PASEF) over an m/z range of 100–1350. Internal mass calibration was achieved using sodium formate and an Agilent low-concentration tuning mix.

Raw data were processed in MetaboScape (version 2026) using the T-Rex 4D feature detection algorithm, integrating m/z, retention time, ion mobility, and intensity information. Metabolite annotation was based on in-house MS^2^ libraries and public databases, including GNPS, LipidBlast, Bruker NIST HRMS, MetaboBase Personal Library 3.0, and HMDB.

For statistical analysis, each sample represents an independent biological replicate of CFS preparation, and replicate-level values were used as independent observations.

All metabolomics experiments were performed with 8 biological replicates per condition. Data were normalized and processed using standard workflows to ensure comparability across samples.

### 2.6. Phylogenetic Analysis of Lr6127

A high-quality bacterial genome of Lr6127 was assembled using Autocycler v0.2.1 [26] by integrating long reads generated with the MinION platform (Oxford Nanopore Technologies, Oxford, UK) and short reads sequenced on the NextSeq1000 sytsem (Illumina, San Diego, CA, USA). The genome consisted of 2,222,416 bp in 2 contigs with a G + C content of 38.7%. Taxonomy was verified by whole-genome average nucleotide identity (ANI), which showed 96.5% nucleotide identity to the type strain DSM 20016, consistent with assignment to the species *L. reuteri.*

### 2.7. Lr6127 Adhesion and Pathogen Exclusion Assays

Lr6127 adhesion: The adhesion assay was performed as previously described by Rasmussen et al. [27]. In brief, MCA-B1 cells were seeded in 96-well plates at a density of 2 × 10^4^ cells/well in a total volume of 0.2 mL and grown for two days until 100% confluency was reached. Lr6127 was grown in MRS at 37 °C under anaerobic conditions for 24 h, the OD was measured at 600 nm, and the concentration was adjusted to an OD of 1.00 (1 × 10^9^ CFU/mL). A 30 μl sample of Lr6127 was then added directly onto the MCA-B1 cells in each well in 96-well plates, and the cells were cocultured for 30 min at 37 °C (CFU_loaded_). A 30 min incubation was used to assess the initial adhesion of Lr6127 and was consistently applied as the first step in exclusion assays to ensure comparability while minimizing confounding effects. The cell monolayer was then washed five times with PBS, and the cells were lysed with cold 0.1% Triton X-100 solution. The lysates were then serially diluted (10-fold) in PBS and plated onto MRS agar for the enumeration of adherent bacteria (CFU_adhered_). Plates were cultured at 37 °C for 24 h before colony counting. To identify the loaded bacteria quantity (CFU_loaded_), the bacterial suspension, prior to its addition to the MCA-B1 cells, was also serially diluted in parallel and plated onto MRS plates. The percentage adhesion of Lr6127 to the MCA-B1 cells was calculated according to the following formula:

Cell adhesion (%) = [(CFU_adhered_)/(CFU_loaded_)] × 100

The data are means from 3 experiments with 5 replicates per experiment.

Pathogen exclusion: The exclusion assay was to determine the capacity of Lr6127 to exclude ETEC, *C. perfringens*, and *S.* Typhimurium from adhering to MCA-B1 cells when applied to the cells before the bacterial application. The assay was performed in a similar manner to that described by Rasmussen et al. [27] but adapted for a study in canine cells. In brief, in the exclusion assay, MCA-B1 cells were seeded into plates at 2 ×10^4^ cells/well and grown to 100% confluency. Fresh cultures of Lr6127 were prepared, with their OD measured and adjusted to 1.00 (1 × 10^9^ CFU/mL), and the bacteria were loaded directly onto MCA-B1 cells, as described for the adhesion assay. The 8 test ETEC, 9 *C. perfringens*, and 5 *S.* Typhimurium isolates (as listed in Supplementary Table S1) were grown in culture media, and the OD of each culture was adjusted to an OD of 1.00 as for the Lr6127 cultures. Preliminary calibration experiments using serial dilution and plate counts indicated that OD_600_ = 1.0 corresponded to approximately 1 × 10^9^ CFU/mL for each bacterial species used. A 300 µL sample of each resulting pathogen culture was then centrifuged; then, the pellet was resuspended in 30 μL of culture medium and the resulting suspension was added directly to the Lr6127 MCA-B1 cell coculture without a washing step. The cells were then incubated at 37 °C for 30 min. After incubation, the MCA-B1 cells were washed (five times with PBS) and lysed with 0.1% Triton-X solution, as before. The lysates were serially diluted (10-fold) and loaded onto either LAMVAB agar plates (MRS agar with 20 mg/L vancomycin for the enumeration of Lr6127, CFU_Lr6127_) or MacConkey plates (for the enumeration of ETEC, CFU_ETEC_). The plates were cultured as for the adhesion assay, the CFUs were counted (CFU _adhered_), and the percentage of adhered cells was calculated, as in the cell adhesion assay.

The reduction in the percentage adhesion (here termed ‘exclusion’) in the Lr6127-treated groups compared to the response in the medium control (MCA-B1 cells with *E. coli* but without Lr6127) was calculated according to the equation below.

The percentage *E. coli* adhered was calculated according to the following equation:

%ETEC_adhered_ (%ETEC_adhered)_ = (CFU_ETEC adhered_/(CFU_ETEC loaded_) × 100

Exclusion (%) = [1 − (%ETEC_adhered, with Lr6127_/%ETEC_adhered, without Lr6127_] × 100

Lr6127’s exclusion of *C. perfringens* or *S.* Typhimurium was assayed using a similar procedure, except that the lysate was placed on a CP ChromoSelect (Merck, Søborg, Denmark) or Chromaga Salmonella for numeration, respectively. The experiments were performed three times with nine replicates in total. For statistical analysis, technical replicates were averaged within each experiment, and isolate-level means derived from independent experiments were used as independent observations.

### 2.8. Wound-Healing Assay

A wound-healing assay was performed using a 2-Well Culture Insert in a 35 mm µ-Dish (Ibidi, Gräfelfing, Germany). Briefly, MCA-B1 epithelial cells were seeded at 6 × 10^5^ cell/mL outside the insert area, allowing for cell attachment and growth around the insert overnight at 37 °C in an atmosphere of 5% CO2 to obtain a confluent cell layer. The insert was then gently removed with sterile tweezers to create a gap. The cell layer was washed with culture medium to remove cell debris and non-attached cells. The cells were then incubated with fresh medium supplemented with CFS from Lr6127 (equivalent to 10^7^/CFU/mL) or with MRS medium (medium control).

Wound closure was monitored using an inverted microscope (Eclipse Ts2R, Nikon, Ramcon, Aarhus, Denmark) with a 4× magnification objective; images were acquired immediately after insert removal on day 0; the wound area was measured in the following 10 days, quantified manually using the Nikon Imaging Software v5.30 (NIS-Elements, Nikon, The Netherlands), and expressed as the area under the curve according to the following: The wound area value was first converted to logarithmic scale (In-scale). Individual data was then normalized using the mean value of the control group as the center. The area under the curve from day 1 to day 10 was expressed by the respective sum and used for comparisons between 2 groups. Briefly, the wound area measurements were log-transformed to stabilize the variance and improve comparability across time points, and normalized relative to the mean of the control group. The area under the curve (AUC) of the normalized wound area over time (days 1–10) was calculated for each biological replicate and used for statistical comparison, with lower AUC values indicating increased wound closure. All experiments were performed with three independent biological replicates (*n* = 3) for each treatment group. For statistical analysis, values derived from each biological replicate were used as independent observations.

### 2.9. Proteomic Profiling of MCA-B1 Cells Following Lr6127 CFS Exposure

MCA-B1 cells were prepared as previously described and grown to full confluence. Fresh culture medium was applied, followed by the addition of Lr6127 cell-free supernatant (CFS) at a concentration corresponding to 1 × 10^7^ CFU/mL. Plates were maintained overnight at 37 °C in a humidified atmosphere containing 5% CO_2_. The control samples received DMEM supplemented with MRS only. Eight biological replicates were included per treatment.

Cells were rinsed with PBS and lysed using a solution containing 5% SDS, 100 mM triethylammonium bicarbonate, and protease inhibitor cocktail (Roche Diagnostics, Merck Life Science, Copenhagen, Denmark). Proteomic analysis was conducted in accordance with the protocol described by Kadekar et al. [28]. Peptide separation and analysis were performed using a Vanquish Neo UHPLC system coupled to a Q Exactive HF mass spectrometer (Thermo Fisher Scientific, Bremen, Germany). A trap-and-elute configuration was employed using a NanoViper trap column and a Waters nanoEase M/Z Peptide BEH C18 analytical column (Waters, Milford, MA, USA), with a 70 min gradient at a flow rate of 2000 nL/min. Data-dependent acquisition with HCD fragmentation was applied.

The raw mass spectrometry data were processed using Proteome Discoverer version 3.0 and searched against the UniProt *Canis familiaris* protein database using the Mascot search engine. Proteomic measurements were conducted using 8 biological replicates. Statistical analysis was performed using biological replicates as independent observations.

### 2.10. Statistical Analysis

For assays other than protein expression, group differences were assessed using the Kruskal–Wallis H test (one-way analysis of variance on ranks), and pairwise comparisons were performed using the Mann–Whitney U test. Technical replicates were averaged within each independent experiment prior to statistical analysis.

For assays involving multiple isolates, isolate-level means derived from independent experiments were used as the unit of analysis. Biological replicates refer to independent experiments performed on different days. Data are presented as mean ± standard error (SE), and results were considered statistically significant at *p* < 0.05.

Protein expression data were analyzed using multivariate latent class analysis (LCA), followed by pairwise comparisons using *t*-tests with a false discovery rate (FDR) threshold of 5%.

## 3. Results

### 3.1. Cell-Free Supernatant from Lr6127 Inhibited the Growth of ETEC, C. perfringens, and S. Typhimurium Isolates

**Figure 1:**
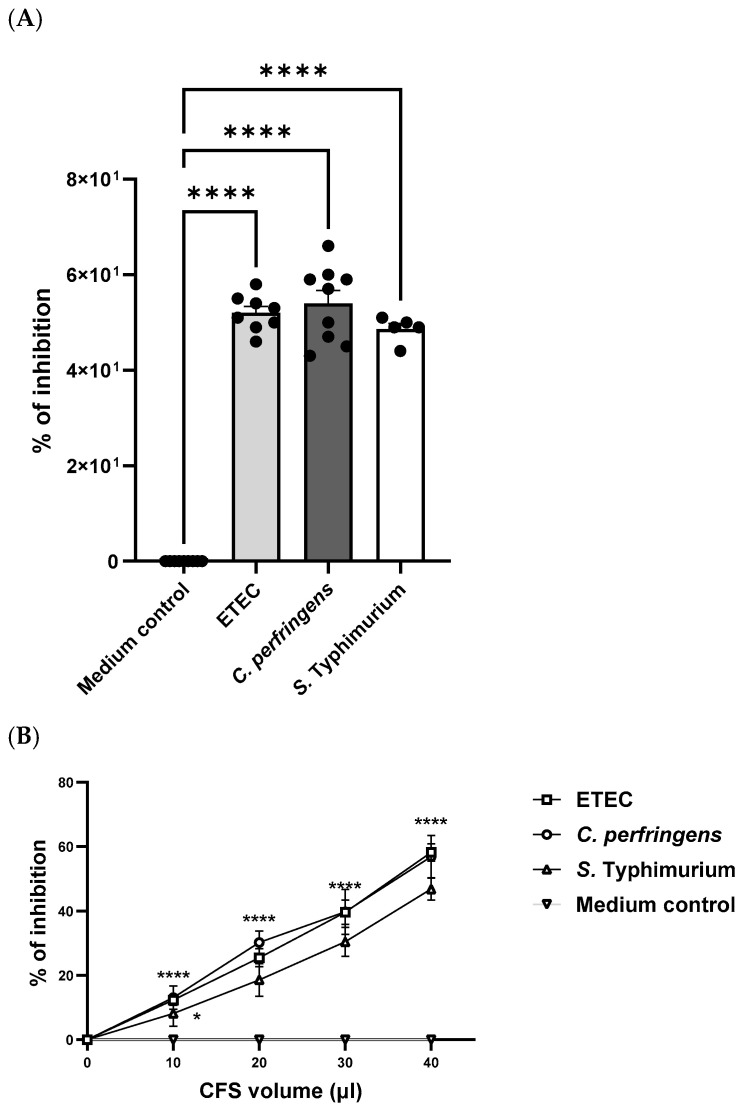
Effect of *Limosilactobacillus reuteri* 6127 (Lr6127) cell-free supernatant (CFS) and medium control on the growth of ETEC, *C. perfringens*, and *S.* Typhimurium isolates. (**A**) Growth inhibition after 16 h of incubation, expressed as percentage inhibition normalized to the medium-only control. (**B**) Growth inhibition increased in a dose-dependent manner with increasing volume of Lr6127 CFS added to the culture. Values are presented as means ± standard errors (SEs). Experiments were performed three times with three technical replicates per experiment. Values represent isolate-level means from independent experiments, with technical replicates averaged within each experiment. Pairwise comparisons were performed relative to the medium control. In panel (**B**), data points at 10–40 µL CFS were compared to the 0 µL control. All comparisons yielded *p* < 0.0001, except for *S.* Typhimurium at 10 µL CFS (*p* < 0.05). * *p* < 0.05; **** *p* < 0.0001.

To assess whether Lr6127 exerted direct antimicrobial effects against clinically relevant canine enteric pathogens, we evaluated the impact of its cell-free supernatant (CFS) on pathogen growth under controlled in vitro conditions. Pathogen growth inhibition was quantified as percentage inhibition normalized to the medium control following 16 h of incubation. No inhibition was observed in the medium control, whereas exposure to Lr6127 CFS resulted in marked suppression of ETEC, *C. perfringens*, and *S.* Typhimurium growth (Figure 1A).

Across all isolates, the average inhibition of ETEC growth was 52.0 ± 1.3%, while *C. perfringens* showed an average inhibition of 54.0 ± 2.7%. The growth of *S.* Typhimurium was inhibited by 48.6 ± 1.2%. The inhibition by Lr6127 CFS was highly significant for all three pathogen groups relative to the medium control (*p* < 0.0001; Figure 1A).

Dose–response analysis further demonstrated that pathogen growth inhibition increased progressively with increasing volume of Lr6127 CFS added to the coculture (Figure 1B). For example, *E. coli* growth inhibition increased from 12.8 ± 0.7% at 10 µL CFS to 58.2 ± 1.0% at 40 µL CFS, illustrating a strong concentration-dependent effect. Similar dose-dependent trends were observed for *C. perfringens* and *S.* Typhimurium, although the inhibition of *S.* Typhimurium remained lower at equivalent CFS volumes. Statistically significant inhibition relative to the medium control was observed at multiple CFS volumes, with significance levels ranging from *p* < 0.05 to *p* < 0.0001, as indicated in Figure 1B.

### 3.2. Antimicrobial and Aromatic Amino Acid-Derived Metabolites in Lr6127 CFS and Corresponding Genomic Features

To identify metabolites that may contribute to the growth-inhibitory effects of Lr6127 CFS observed in pathogen inhibition assays (Figure 1), untargeted metabolomic profiling was performed on Lr6127 CFS and the corresponding medium control.

**Figure 2:**
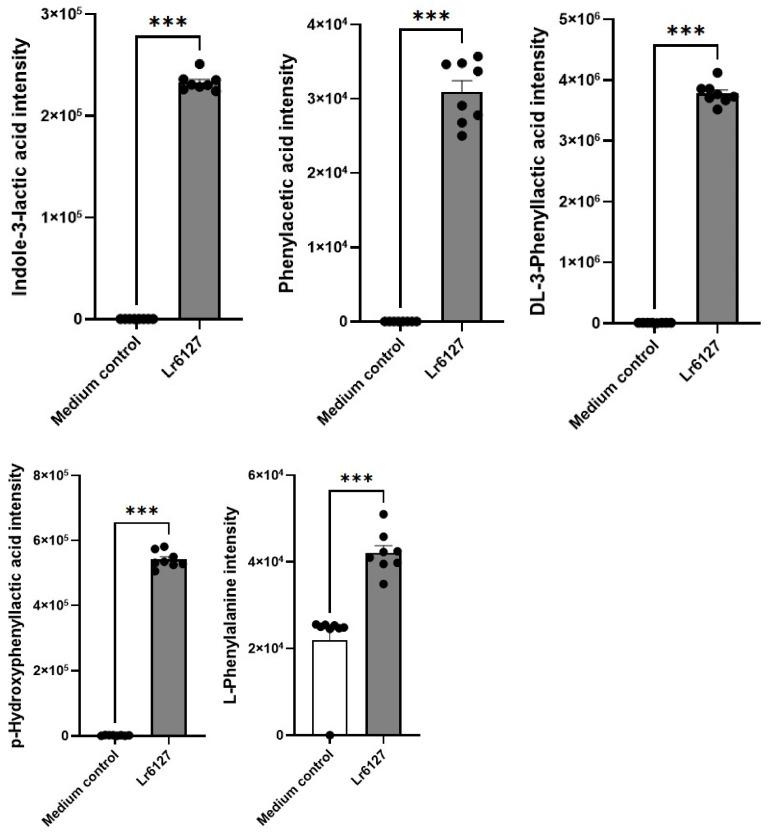
Abundance of antimicrobial-associated metabolites in CFS from Lr6127 determined by untargeted metabolomics. Indole-3-lactic acid, phenylacetic acid, DL-3-phenyllactic acid, p-hydroxyphenyl lactic acid, and L-phenylalanine are shown. Data are presented as means ± standard errors (SEs) from eight replicates per treatment. Each replicate represents an independent CFS preparation and analysis. Statistical significance was determined by comparison with the medium control. *** *p* < 0.001.

Several antimicrobial-associated metabolites were significantly enriched in Lr6127 CFS compared with the medium control (Figure 2). Indole-3-lactic acid, an indole-derived tryptophan metabolite commonly produced by lactic acid bacteria, was detected at high abundance in Lr6127 CFS, whereas it was not detected in the medium control (*p* < 0.001). Phenylacetic acid, an aromatic organic acid derived from phenylalanine metabolism, was likewise absent from the medium control and present at 3.1 ± 0.1 × 10^4^ in Lr6127 CFS (*p* < 0.001).

DL-3-phenyllactic acid, an aromatic lactic acid derivative frequently associated with microbial fermentation, showed marked enrichment in Lr6127 CFS, compared with a low background level in the medium control (559-fold, *p* < 0.001). Similarly, p-hydroxyphenyllactic acid, a hydroxylated aromatic lactic acid commonly linked to microbial metabolism, was significantly elevated in Lr6127 CFS (*p* < 0.001). L-phenylalanine, an essential aromatic amino acid and central precursor in phenyl-derived metabolism, was also present at higher abundance in Lr6127 CFS relative to the medium control (*p* < 0.001).

A comprehensive list of significantly altered metabolites between the Lr6127 CFS and the medium control is provided in Supplementary Figure S1 and Supplementary Excel Metabolomics sheet.

Alongside the enrichment of aromatic amino acid-derived metabolites detected in the Lr6127 cell-free supernatant (CFS), genomic analysis identified adjacent aromatic amino acid aminotransferase (ArAT) and aromatic lactate dehydrogenase (ALDH) homologs within a single gene cluster in the Lr6127 genome, indicative of roles for these genes in the classical aromatic amino acid catabolism pathway, the high activity of which is consistent with the elevated abundance of all detected metabolites, except L-phenylalanine.

### 3.3. Lr6127 Colony-Forming Units Excluded Pathogen Adhesion to MCA-B1 Cells

Following the identification of antimicrobial-associated metabolites in Lr6127 cell-free supernatant, we next examined whether viable Lr6127 affected pathogen interactions at the epithelial surface. In a preliminary assay, the binding of Lr6127 to MCA-B1 cells was observed following 30 min of coculture, with an average attachment of 2.0 ± 0.3%. Based on this interaction, adhesion exclusion assays were subsequently performed to determine whether viable Lr6127 could interfere with pathogen attachment to host epithelial cells. MCA-B1 cells were pre-treated with Lr6127 colony-forming units (CFUs) prior to pathogen exposure, and pathogen adhesion was quantified as percentage exclusion relative to the medium control condition in which MCA-B1 cells were exposed to pathogens in the absence of Lr6127.

**Figure 3:**
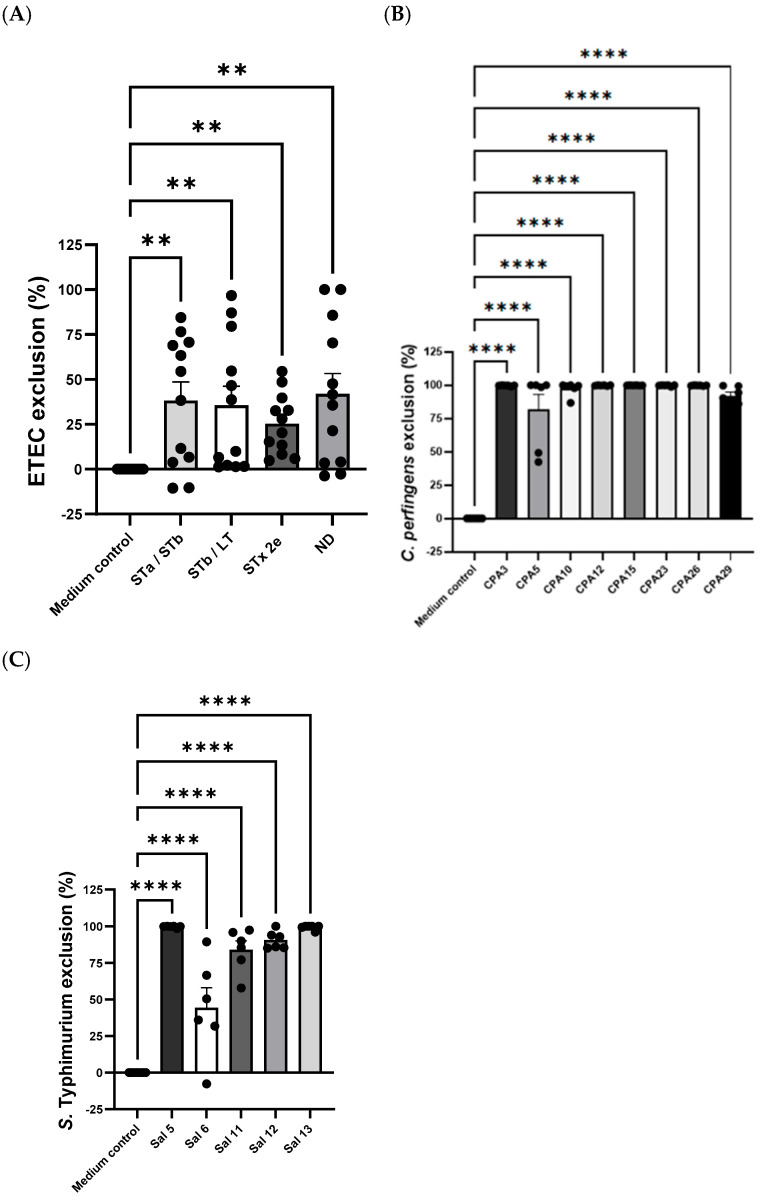
Percentage exclusion of pathogen adhesion to MCA-B1 cells following pre-treatment with Lr6127. (**A**) Exclusion of ETEC isolates representing heat-stable enterotoxin a/b (STa/STb), heat-stable enterotoxin b/heat-labile enterotoxin (STb/LT), Shiga toxin 2e (STx2e), and non-defined (ND) subtypes. (**B**) Exclusion of individual *C. perfringens* isolates. (**C**) Exclusion of individual *S.* Typhimurium isolates. Data are presented as means ± standard errors (SEs) and were obtained from three independent experiments with six technical replicates per isolate. Values represent isolate-level means, with technical replicates averaged within each experiment. Statistical comparisons were performed relative to the control condition (MCA-B1 cells exposed to pathogens in the absence of Lr6127). ** *p* < 0.01; **** *p* < 0.0001.

Pre-treatment with Lr6127 significantly reduced the adhesion of ETEC isolates representing multiple toxin subtypes to MCA-B1 cells (Figure 3A). The mean exclusion of STa/STb isolates, which express heat-stable enterotoxins a and b (STa, STb), was 38.2 ± 10.3%, while STb/LT isolates, which express heat-stable enterotoxin b and heat-labile enterotoxin (LT), showed a mean exclusion of 35.5 ± 10.6%. The adhesion of STx2e isolates, which produce Shiga toxin 2e (STx2e), was reduced by 25.3 ± 4.8%. Non-defined (ND) ETEC isolates exhibited the highest exclusion, averaging 42.0 ± 11.3%. All E. coli subtypes displayed significantly reduced adhesion relative to the control condition (*p* < 0.01; Figure 3A).

Lr6127 pre-treatment resulted in pronounced exclusion of *C. perfringens* (A type) adhesion across all tested isolates (Figure 3B). Near-complete exclusion was observed for isolates CPA3, CPA12, CPA15, CPA23, and CPA26, with mean exclusion values ranging from 99.7 ± 0.2% to 100.0 ± 0.0%. CPA10 also exhibited strong exclusion (97.3 ± 2.1%). While exclusion of CPA5 and CPA29 was more variable, mean values remained high at 81.7 ± 11.4% and 91.8 ± 3.1%, respectively. All *C. perfringens* isolates showed highly significant reductions in adhesion compared with the control condition (*p* < 0.0001; Figure 3B).

Similarly, adhesion of *S.* Typhimurium isolates to MCA-B1 cells was significantly reduced following Lr6127 pre-treatment (Figure 3C). Near-complete exclusion was observed for isolate Sal 5 (99.8 ± 0.2%) and Sal 13 (99.2 ± 0.6%). Sal 11 and Sal 12 exhibited substantial exclusion, averaging 84.0 ± 6.0% and 90.6 ± 2.5%, respectively. In contrast, Sal 6 displayed greater variability, with a mean exclusion of 44.5 ± 13.5%. Despite this variability, all *S.* Typhimurium isolates showed statistically significant reductions in adhesion relative to the control (*p* < 0.0001; Figure 3C).

The observation that pathogen exclusion occurred despite relatively modest Lr6127 adhesion (~2%) suggests that adhesion and exclusion are not directly proportional. This finding indicates that adhesion alone may not fully predict exclusion efficacy under the conditions tested.

### 3.4. Cell-Free Supernatant from Lr6127 Facilitated Wound Healing

**Figure 4:**
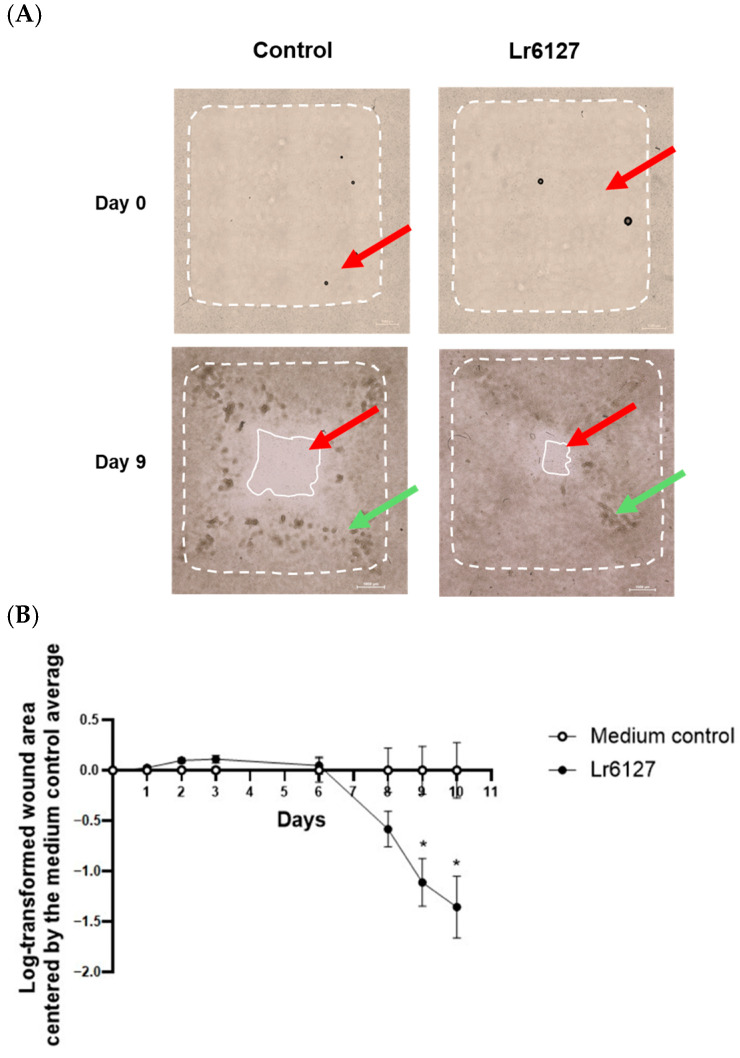
Effect of Lr6127 cell-free supernatant (CFS) on wound healing in MCA-B1 cells. (**A**) Representative images showing reduced wound area (red arrows indicate representative locations within the wound area) in Lr6127 CFS-treated MCA-B1 monolayers compared with the MRS medium control on day 9 (dashed square). Green arrows indicate regions of epithelial healing. (**B**) Wound closure kinetics expressed as the cumulative area under the curve of normalized wound area values. Wound area data was log-transformed and normalized using the mean of the medium control group as the center. Values for days 1–10 were summed for each biological replicate and used for comparison between groups. Data represent three independent replicates per treatment. Values derived from each biological replicate were used as independent observations. Pairwise comparisons with the medium control were performed, and statistically significant differences were observed on both days 9 and 10. * *p* < 0.05.

Because pathogen adhesion represents an early step in epithelial barrier disruption, we next examined whether Lr6127 cell-free supernatant (CFS) influenced subsequent epithelial wound repair. The effect of Lr6127 cell-free supernatant (CFS) on epithelial wound repair was assessed using a wound-healing assay on MCA-B1 cells, and wound closure was monitored over a 10-day culture period. Representative images showed comparable wound areas at day 0, whereas a visibly reduced wound area was observed in CFS-treated monolayers compared with the medium control by day 9 (Figure 4A).

The wound closure kinetics were quantitatively analyzed using the cumulative area under the curve (AUC) of log-transformed and normalized wound area values over time using the medium control as the center. When summed from days 1 to 9, the cumulative wound area was −0.0 ± 0.6 for the medium control and −1.4 ± 0.6 for Lr6127 CFS-treated cells, indicating significantly enhanced wound closure by day 9. Lower cumulative AUC values reflect greater wound closure over time. Inclusion of day 10 further strengthened this effect, with summed wound area values of −0.0 ± 0.9 for the control group and −2.8 ± 0.9 following CFS treatment (Figure 4B). Pairwise statistical comparisons demonstrated that the wound area reduction in the Lr6127 CFS-treated group was significant at both days 9 and 10 relative to the medium control (*p* < 0.05; Figure 4B). The full wound area measurements underlying the normalized AUC analysis are provided in Supplementary Table S2.

### 3.5. Proteomic Identification of Wound-Healing Proteins in MCA-B1 Cells Cultured with Lr6127 CFS

**Figure 5:**
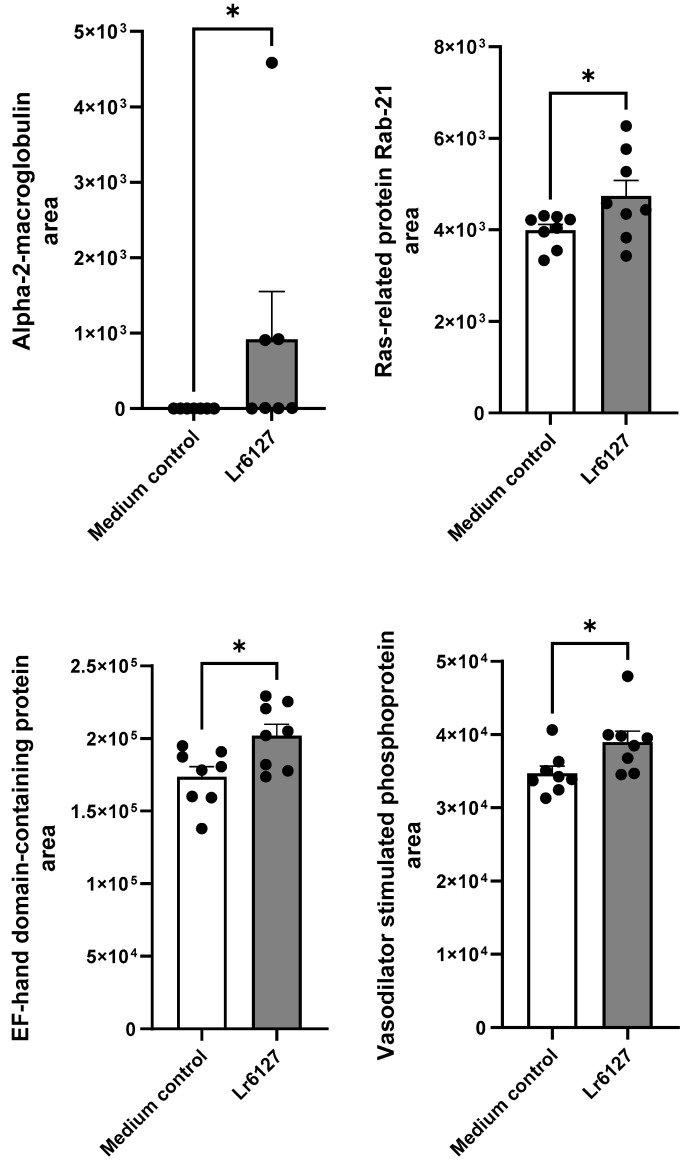
Abundance of wound-healing-related proteins in MCA-B1 cells following exposure to Lr6127 cell-free supernatant (CFS). Protein levels of alpha-2-macroglobulin, Ras-related protein Rab-21, EF-hand domain-containing protein, and vasodilator-stimulated phosphoprotein were quantified in MCA-B1 cells cultured with Lr6127 CFS or medium control. Data are presented as means with associated standard error (SE) bars. The experiment was performed with eight replicates per treatment group, which were used as independent observations for statistical analysis. * *p* < 0.05.

Building on the observed enhancement of epithelial wound closure induced by Lr6127 CFS in MCA-B1 cells, we next examined whether this functional response was accompanied by changes in the abundance of proteins known to participate in tissue remodeling, cytoskeletal organization, and cell migration. To this end, the abundance of wound-healing-related proteins was quantified in MCA-B1 cells following exposure to Lr6127 CFS. The complete proteomics dataset, including relative protein abundances, fold changes, and statistical analysis, is provided in the Supplementary Excel Proteomics sheet, while selected proteins with established relevance to epithelial remodeling and wound healing are presented in Figure 5.

Treatment with Lr6127 CFS resulted in a pronounced and significant increase in alpha-2-macroglobulin, a multifunctional protease inhibitor that contributes to extracellular matrix remodeling during tissue repair. The protein abundance increased from 2.3 ± 0.2 in the medium control group to 9.2 ± 1.9 × 10^2^ following CFS treatment (*p* < 0.05).

Similarly, the abundance of the Ras-related protein Rab-21, which plays a role in vesicular trafficking and integrin-mediated cell migration, was significantly elevated in response to CFS treatment, increasing from 4.0 ± 0.1 × 10^3^ in the medium control to 4.7 ± 0.1 × 10^3^ (*p* < 0.05). Proteins involved in the calcium-dependent regulation of cytoskeletal dynamics were likewise affected, with the EF-hand domain-containing protein showing a moderate but significant increase from 1.7 ± 0.1 × 10^5^ to 2.0 ± 0.1 × 10^5^ following CFS exposure (*p* < 0.05). In addition, vasodilator-stimulated phosphoprotein, a key mediator of actin filament assembly and directional cell migration, was significantly increased compared with the medium control, rising from 3.5 ± 0.1 × 10^4^ to 3.9 ± 0.2 × 10^4^ (*p* < 0.05).

## 4. Discussion

This study provides in vitro evidence from a controlled canine epithelial model that *Limosilactobacillus reuteri* ATCC PTA 6127 (Lr6127) is associated with effects relevant to epithelial barrier function. Using this model, we show that Lr6127 was associated with reduced pathogen growth and adhesion, and increased wound closure through coordinated changes in wound-healing-associated proteins. Together, these findings are consistent with multiple probiotic-associated effects in vitro [29,30].

Lr6127 cell-free supernatant (CFS) significantly inhibited the growth of ETEC, *C. perfringens*, and *S.* Typhimurium in a dose-dependent manner, indicating that antimicrobial activity scales with metabolite exposure rather than reaching a fixed threshold. These pathogens represent distinct modes of epithelial disruption in dogs, including toxin-mediated secretory or cytotoxic damage by *E. coli* [3,31], toxin-driven epithelial injury associated with *C. perfringens* type A [32,33], and epithelial invasion and inflammatory signaling induced by *S.* Typhimurium [34]. The consistent inhibition observed across these biologically diverse pathogens is consistent with a broad antimicrobial effect relevant to canine gastrointestinal function.

Untargeted metabolomic profiling identified multiple antimicrobial-associated metabolites that are enriched in Lr6127 CFS, including indole-3-lactic acid (ILA), DL-3-phenyllactic acid (PLA), p-hydroxyphenyllactic acid (p-HPLA), and phenylacetic acid. ILA, an indole-derived tryptophan metabolite produced by lactic acid bacteria, has been shown to inhibit *E. coli* growth and affect epithelial barrier responses via host signaling pathways [3,35]. PLA and p-HPLA exhibit broad antimicrobial activity against Gram-negative enteropathogens and *Clostridium* species through environmental acidification and membrane perturbation [36,37], while phenylacetic acid has been reported to interfere with bacterial metabolism under gut-relevant conditions [38]. Importantly, no single metabolite detected in Lr6127 CFS is known to fully explain the breadth or magnitude of inhibition observed. Instead, the concurrent presence of metabolites with complementary antimicrobial modes of action, together with the proportional dose–response pattern, is consistent with a contribution from the combined activity of the metabolite pool rather than by a single dominant compound. For example, acid-mediated membrane perturbation by PLA and p-HPLA may plausibly increase bacterial susceptibility to indole-derived metabolites such as ILA, supporting a potential additive or synergistic antimicrobial effect at the biological level [35,36,37]. These findings support a meaningful contribution of the metabolite profile to the observed antimicrobial effects, while the relative roles of individual components represent an interesting avenue for further investigation.

To place the observed aromatic amino acid-derived metabolite profile of Lr6127 in a broader biological context, it is useful to consider the metabolic and genomic features associated with aromatic amino acid catabolism in lactic acid bacteria [39]. The conversion of aromatic amino acids into indole- and phenyl-derived metabolites in lactic acid bacteria has been described to proceed through a multistep pathway that is largely inferred from comparative genomics and metabolomics [39,40]. To date, only two enzymes involved in this process have been experimentally characterized in lactic acid bacteria: an aromatic amino acid aminotransferase (ArAT), which catalyzes the initial transamination of aromatic amino acids to their corresponding aromatic pyruvic acids, and an aromatic lactate dehydrogenase (ALDH), which reduces these intermediates to aromatic lactic acids [36,37]. In this context, the identification of adjacent ArAT and ALDH homologs within the Lr6127 genome provides biological context for the enrichment of aromatic amino acid-derived metabolites observed in the cell-free supernatant. Although transcriptional regulation and enzymatic activity were not assessed in the present study, this genomic organization is consistent with previously described metabolic frameworks in lactic acid bacteria and may contribute to the focused aromatic metabolite profile detected for Lr6127 [39]. In addition to these epithelial-intrinsic mechanisms, bacterially derived indole metabolites such as indole-3-lactic acid have been reported to engage aryl hydrocarbon receptor (AhR) signaling and have been associated with epithelial barrier effects in other experimental systems [39,41]. While AhR-dependent responses were not directly assessed here, the presence of ILA in Lr6127 CFS provides relevant biological context for the barrier-supportive effects observed in this study.

Beyond limiting pathogen growth, Lr6127 provided a second defensive layer by significantly reducing the adhesion of multiple ETEC toxin subtypes, *C. perfringens* type A isolates, and *S.* Typhimurium to canine epithelial cells. Because epithelial attachment is a prerequisite for toxin delivery and invasion, particularly for enterotoxigenic and invasive pathogens, this exclusion effect likely contributes meaningfully to intestinal defense [29,34]. The observation that pathogen exclusion occurred despite relatively modest Lr6127 adhesion suggests that adhesion and exclusion are not directly proportional. Similar patterns have been reported in other probiotic systems, where low adhesion (≈0.2–3%) can still be associated with high exclusion efficiency, while higher adhesion does not necessarily result in stronger exclusion [27]. In the present study, adhesion was only assessed for Lr6127, and corresponding data are not available for all pathogens; therefore, pathogen-specific adhesion properties (e.g., for *C. perfringens* and *S.* Typhimurium) may contribute to the observed variability.

This may reflect that even limited or transient epithelial interactions and competition for binding sites are sufficient to impair early pathogen attachment, although the precise mechanisms were not directly examined in the current study. Compared with spore-forming *Bacillus* strains previously evaluated in porcine epithelial models, Lr6127 appears to reduce pathogen adhesion through early interactions at the epithelial interface rather than through extensive or persistent colonization [27].

Importantly, suppression of pathogen pressure was accompanied by enhanced epithelial wound healing, with statistically significant effects emerging only at later time points. This delayed response indicates that Lr6127 CFS may be associated with sustained epithelial remodeling and barrier restoration processes, which are characteristic of mature mucosal repair rather than rapid, transient closure [42,43]. Rather than acting independently, the proteins modulated by Lr6127 CFS may be interpreted as components of a coordinated epithelial response associated with wound healing. Increased alpha-2-macroglobulin may contribute to stabilizing the extracellular wound microenvironment by limiting excessive protease activity, thereby supporting the maintenance of a provisional matrix scaffold required for effective integrin-dependent migration [44]. Within this context, the concurrent upregulation of vasodilator-stimulated phosphoprotein (VASP) is consistent with processes involved in epithelial migration, although functional coupling was not directly assessed [42,43]. The Ras-related protein Rab-21 represents a potential link between cytoskeletal remodeling and adhesion dynamics by regulating integrin trafficking, thereby potentially contributing to the coordination of cell movement with adhesion turnover and subsequent re-anchoring [45]. The concomitant increase in an EF-hand domain-containing calcium-binding protein further suggests a possible involvement of calcium-dependent coordination of these processes, potentially facilitating the transition from active migration to junctional reassembly and barrier stabilization, which may be consistent with the delayed yet sustained wound-healing response observed [46]. In addition, prior studies on *L. reuteri* reported broader host-associated pathways, including oxytocin-linked signaling, that may support tissue repair in vivo [47], providing complementary context for the wound-healing effects observed here. These findings should be interpreted within the study scope, as only a subset of proteins was analyzed without pathway-level or post-translational validation. Thus, the results remain indicative, and epithelial barrier integrity was not directly assessed. Future studies will be needed to further substantiate these findings.

When considered collectively, the combined effects of pathogen growth inhibition, adhesion exclusion, and epithelial repair are consistent with a model in which Lr6127 is associated with multiple pathogen- and epithelial-related effects. The direct suppression of pathogen proliferation and epithelial colonization reduces the luminal and surface-associated pathogen burden, while enhanced wound healing preserves epithelial continuity and limits microbial translocation, a key driver of intestinal inflammation [3]. Rather than acting through a single dominant pathway, Lr6127 integrates antimicrobial metabolite production with epithelial barrier-related responses, a layered set of antimicrobial and epithelial-related effects that may be relevant to the dynamic and stress-responsive canine gut environment [1].

This study contributes contextual insight into the associations between Lr6127 and epithelial defense and repair in a canine-relevant cell model, thereby providing a basis for further translational investigation. The use of a two-dimensional canine epithelial system enables the controlled examination of host–microbe interactions and probiotic-mediated effects, although it does not fully capture the complexity of the intestinal microenvironment, including the mucus layers, commensal microbiota, immune cell contributions, and mechanical forces present in vivo. Pathogen inhibition was assessed under defined in vitro conditions using a representative panel of clinically relevant enteric pathogens, allowing specific associations to be drawn between microbial metabolites and epithelial responses. While the upstream signaling pathways linking Lr6127-derived metabolites to epithelial regulatory networks remain incompletely characterized, the coordinated antimicrobial, adhesion-exclusion, and wound-healing responses observed in this study are consistent with biologically relevant probiotic activity. Further investigation using more physiologically complex systems, such as canine intestinal organoids and well-designed in vivo studies, will be necessary to confirm translational relevance, refine dosing strategies, and evaluate long-term safety and efficacy. The integration of multi-omics approaches in such models may further elucidate how microbial metabolites, epithelial signaling pathways, and immune modulation interact to produce the coordinated probiotic effects described here.

## 5. Conclusions

*Limosilactobacillus reuteri* ATCC PTA 6127 was associated with pathogen growth inhibition, reduced epithelial adhesion, and enhanced epithelial wound closure in a canine epithelial model. These findings provide a coherent characterization of the antimicrobial and epithelial-associated responses. Further validation in additional experimental systems will be valuable to confirm the robustness and broader applicability of these findings, and to support their translation across different biological settings.

## Data Availability

The original contributions presented in this study are included in the article/Supplementary Material. Further inquiries can be directed to the corresponding author.

## Supplementary Materials

The following supporting information can be downloaded at: https://www.mdpi.com/article/10.3390/microorganisms14071422/s1, Supplementary Table S1: Identification virulence profiles, and host origin of the Enterotoxigenic *Escherichia coli*, *Clostridium perfringens* and *Salmonella enterica serovar Typhimurium* isolates used in the assays. Supplementary Figure S1: Heatmap of selected metabolites across sample groups. Supplementary Table S2: Wound area (mm^2^) measurements for MCA-B1 cells treated with Lr6127 CFS or control medium over the experimental time course. Supplementary Excel Sx: Proteomics dataset. Relative protein abundance, fold changes, and statistical significance for wound-healing–associated proteins in MCA-B1 cells treated with Lr6127 cell-free supernatant (CFS) compared with MRS control. The dataset comprises 8 replicates per treatment and 2659 quantified proteins. Statistical analysis was performed using a multivariate latent class model (LCA), with treatment effects expressed as latent Z-scores and false discovery rate (FDR)-adjusted *p*-values (5%). Mean (SEM) values and fold changes are provided in the MEANS sheet. Protein annotation was performed using DAVID based on Gene Ontology information. Proteins associated with wound-healing processes were curated based on annotation and literature and are listed in a dedicated sheet.

## Abbreviations

The following abbreviations are used in this manuscript:

A2M	Alpha-2-macroglobulin
*C*. *perfringens*	*Clostridium perfringens*
CFS	Cell-free supernatant
CFU	Colony-forming unit
EF-hand	EF-hand domain-containing protein
ETEC	Enterotoxigenic *Escherichia coli*
ILA	Indole-3-lactic acid
*L. reuteri*	*Limosilactobacillus reuteri*
LT	Heat-labile enterotoxin
MYL6B	Myosin light chain 6B
OD	Optical density
PLA	Phenyllactic acid
p-HPLA	p-Hydroxyphenyllactic acid
Rab21	Ras-related protein Rab-21
*S.* Typhimurium	*Salmonella enterica* subsp. *enterica* serovar Typhimurium
SE	Standard error
STa	Heat-stable enterotoxin a
STb	Heat-stable enterotoxin b
STx2e	Shiga toxin 2e
VASP	Vasodilator-stimulated phosphoprotein
