# Supplementary material for: Evaluation of Limosilactobacillus reuteri ATCC PTA 6127 Reveals Multilayered Antimicrobial and Epithelial Barrier-Supportive Effects in a Canine Epithelial Model
Source: Microorganisms. 2026 Jun 29;14(7):1422. doi: 10.3390/microorganisms14071422 (PMC13413866; doi:10.3390/microorganisms14071422)
Supplement: Supplementary file 1 [file microorganisms-14-01422-s001.zip › Lr6127 pathogen supplementary V5.pdf]

Supplementary Table S1. Identification virulence profiles, and host origin of the Enterotoxigenic *Escherichia coli*, *Clostridium perfringens* and *Salmonella enterica serovar Typhimurium* isolates used in the assays.

IFF Collection Code	Toxin Profile Classification (as used in manuscript)	Animal origin	Country of Isolation	Stb	Sta	K99	LT	F18	987P	K88	F41	Stx2e
EC-2	STa/STb	Swine	Germany	1	1	0	0	0	0	1	0	0
EC-23	STb/LT	Swine	Germany	1	0	0	1	1	0	0	0	0
EC-61	STx2e	Swine	Germany	0	0	0	0	1	0	0	0	1
EC-62	STx2e	Swine	Germany	0	0	0	0	1	0	0	0	1
EC-63	STa/STb	Swine	Germany	1	1	0	0	1	0	0	0	0
EC-88	STb/LT	Swine	Germany	1	0	0	1	0	0	1	0	0
EC-90	Above toxin genes not deteced	Swine	Germany	0	0	0	0	0	0	0	0	0
EC-91	Above toxin genes not deteced	Swine	Germany	0	0	0	0	0	0	0	0	0

In this table, “1” indicates presence, and “0” indicates absence.

IFF Collection Code	Toxin type and Virulence Profile (as used in manuscript)	Animal origin	Country of Isolation
CPA3	A, ≤4 NE, cpb2-, netB-	Swine	Germany
CPA5	A, ≤4 NE, cpb2+, netB-	Swine	Germany
CPA10	A, 8-16 NE, cpb2-, netB-	Swine	Germany
CPA12	A, 8-16 NE, cpb2-, netB-	Swine	Germany
CPA15	A, 8-16 NE, cpb2+, netB-	Swine	Germany

CPA21	A, 32 NE, cpb2+, netB-	Swine	Germany
CPA23	A, ≥64 NE, cpb2-, netB-	Swine	Germany
CPA26	A, ≥64 NE, cpb2-, netB-	Swine	Germany
CPA29	A, ≥64 NE, cpb2+, netB-	Swine	Germany

**IFF Collection Code**	**Serovar Classificatio (classification in manuscript)**	**Animal origin**	**Country of Isolation**
Sal5	Typhimurium	Swine	Germany
Sal6	Typhimurium	Swine	Germany
Sal11	Typhimurium	Swine	The Netherlands
Sal12	Typhimurium	Swine	The Netherlands
Sal13	Typhimurium	Swine	The Netherlands

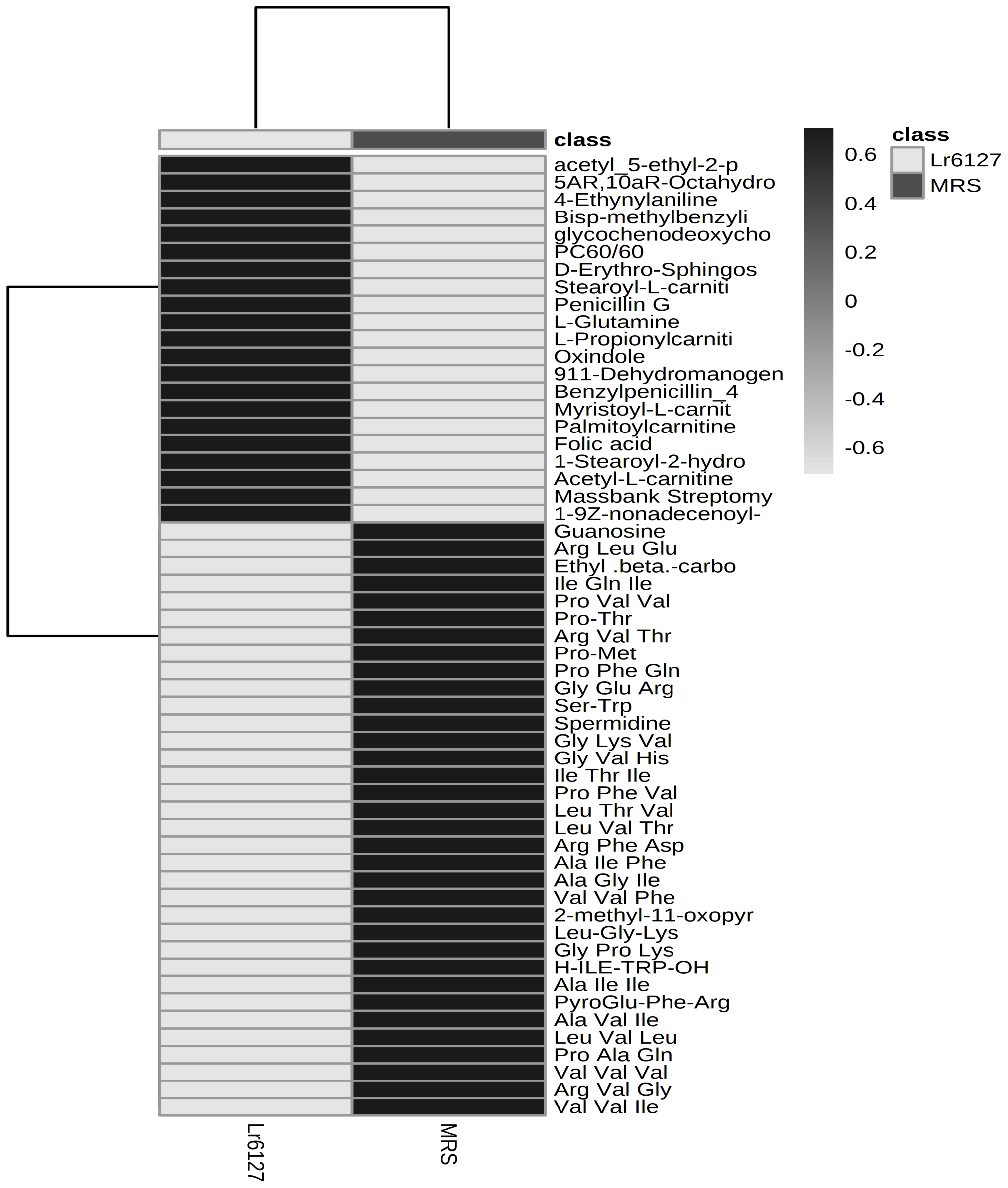
class
acetyl_5-ethyl-2-p
5AR,10aR-Octahydro
4-Ethynylaniline
Bisp-methylbenzyli
glycochenodeoxycho
PC60/60
D-Erythro-Sphingos
Stearoyl-L-carniti
Penicillin G
L-Glutamine
L-Propionylcarniti
Oxindole
911-Dehydromanogen
Benzylpenicillin_4
Myristoyl-L-carnit
Palmitoylcarnitine
Folic acid
1-Stearoyl-2-hydro
Acetyl-L-carnitine
Massbank Streptomy
1-9Z-nonadecenoyl-
Guanosine
Arg Leu Glu
Ethyl .beta.-carbo
Ile Gln Ile
Pro Val Val
Pro-Thr
Arg Val Thr
Pro-Met
Pro Phe Gln
Gly Glu Arg
Ser-Trp
Spermidine
Gly Lys Val
Gly Val His
Ile Thr Ile
Pro Phe Val
Leu Thr Val
Leu Val Thr
Arg Phe Asp
Ala Ile Phe
Ala Gly Ile
Val Val Phe
2-methyl-11-oxopyr
Leu-Gly-Lys
Gly Pro Lys
H-ILE-TRP-OH
Ala Ile Ile
PyroGlu-Phe-Arg
Ala Val Ile
Leu Val Leu
Pro Ala Gln
Val Val Val
Arg Val Gly
Val Val Ile
Lr6127
MRS
0.6
0.4
0.2
0
-0.2
-0.4
-0.6
class
Lr6127
MRS

Supplementary Figure S1. Heatmap of selected metabolites across sample groups.
The plot shows z-score–normalized relative abundances of the 55 most significantly different metabolites between MRS and Lr6127 samples. Metabolites are presented in rows and sample groups in columns. Color intensity reflects relative abundance, with darker shades indicating higher levels. Visualization was generated using MetaboAnalyst 4.0.

The heatmap was generated using annotated metabolites that were identified as significantly different between groups based on univariate statistical analysis using *p*-value (FDR) criteria. Several amino acids and short peptides also exhibited coordinated changes, pointing to systematic variation in amino acid and peptide metabolisms between MRS medium and Lr6127. Please see full dataset in Supplementary Excel Metabolomics sheet.

Supplementary Table S2. Wound area (mm^2) measurements for MCA-B1 cells treated with Lr6127 CFS or control medium over the experimental time course.

Treatment	Day 0	Day 1	Day 2	Day 3	Day 6	Day 8	Day 9	Day 10
Control medium	69.64006	43.68234	28.85607	22.88491	6.851821	1.843687	1.730972	1.459741
Control medium	69.82358	44.11103	28.67036	23.00852	9.642866	3.723446	3.627846	3.339397
Control medium	70.8726	46.27649	30.746	25.10295	9.913843	3.455835	3.43851	3.360782
L. reuteri PTA6127	70.14322	44.52791	30.86518	24.48887	7.925352	1.220102	0.598875	0.355529
L.reuteri PTA6127	69.83786	45.73282	33.37643	27.67723	10.64535	2.226674	1.351397	0.951943
L. reuteri PTA6127	68.58099	47.19754	33.05493	27.07272	8.926337	1.512446	0.94491	0.824898

Each row represents a biological replicate, and values correspond to wound area at the indicated time points. These data form the basis of the normalized AUC analyses presented in the main manuscript.

Supplementary Excel Sx. Proteomics dataset. Relative protein abundance, fold changes, and statistical significance for wound-healing–associated proteins in MCA-B1 cells treated with Lr6127 cell-free supernatant (CFS) compared with MRS control. The dataset comprises 8 replicates per treatment and 2659 quantified proteins. Statistical analysis was performed using a multivariate latent class model (LCA), with treatment effects expressed as latent Z-scores and false discovery rate (FDR)-adjusted p-values (5%). Mean (SEM) values and fold changes are provided in the MEANS sheet. Protein annotation was performed using DAVID based on Gene Ontology information. Proteins associated with wound-healing processes were curated based on annotation and literature and are listed in a dedicated sheet.
